# Hospital–Physician Integration and the Comprehensive Care for Joint Replacement Program

**DOI:** 10.1177/10775587261468837

**Published:** 2026-09-28

**Authors:** Brady Post, Alexandra Harris, Farbod Alinezhad, Gary Young

**Affiliations:** 1 Northeastern University, Boston, MA, USA; 2 Medicare Payment Advisory Commission, Washington, DC, USA; 3 Analysis Group, Boston, MA, USA

**Keywords:** hospital employment of physicians, physician practice organization, Medicare payment policy

## Abstract

We examined how hospital–physician integration influences hip and knee osteoarthritis treatment under different payment models. We leveraged a natural experiment from Medicare’s random assignment of metropolitan statistical areas (MSAs) to the Comprehensive Care for Joint Replacement (CJR) program. In the 12 months after diagnosis, CJR was associated with more primary care physician and orthopedist visits (0.14 and 0.23, *p* < .001 for both), while integration was associated with fewer (−0.16 and −0.59, *p* < .001 for both). We discovered an extensive margin effect: CJR was associated with lower likelihood of joint replacements (−1.9 percentage points [pp], *p* < .001); integration was associated with higher likelihood (1.0 pp, *p* < .001). Among knee replacement patients, those in CJR MSAs were less likely to receive surgery in outpatient settings (−4.6 pp, *p* < .001). Payment models and organizational structure may create opposing incentives, where bundled payment decreases and vertical integration increases the likelihood of joint replacement surgery.

## Introduction

Hip and knee replacements are by far the most popular elective surgeries among older adults. Over half a million are performed annually on Medicare patients, with even greater use projected over the next decade (Shichman et al., 2023). At the same time, orthopedic care, much like other specialties, has become more closely integrated with hospitals; more orthopedic surgeons (hereafter, orthopedists) than at any other time in history are hospital employees rather than independent practitioners (Machta et al., 2020). However, orthopedic care has been comparatively overlooked in recent studies of hospital–physician integration.

Despite growing literature over the last decade that hospital–physician integration generally results in higher health care costs with questionable value to patients, two prominent gaps in our understanding remain (Harris et al., 2025; Jung et al., 2019; Richards et al., 2022; Whaley et al., 2021; Young et al., 2021). First, in the context of integration, orthopedic care is one of the comparatively less studied specialties. This is curious for several reasons, above all because orthopedic care is well known to be a lucrative, high-volume service line for hospitals – one that cross-subsidizes service lines like psychiatry that cut into hospitals’ profit margins (David et al., 2014). Employment of orthopedists may therefore be of special strategic importance to hospitals as they seek to secure orthopedic referrals.

At the same time, physician behavior has been shown to be malleable; a physician’s organization, peer group, and financial incentives influence treatment style, particularly when there is latitude for clinical discretion (Post et al., 2023; Reschovsky et al., 2015; Young et al., 2023). Orthopedists in fee-for-service already face incentives to perform surgeries, as procedures tend to generate more revenue than conservative care. Hospital–physician integration, however, may amplify those incentives further. In a typical knee replacement episode, for example, the Medicare physician fee for the surgeon is about $1,200, but the payment to the hospital is about $13,000 (Centers for Medicare & Medicaid Services, n.d.-a, n.d.-b). Since hospitals gain facility fees and downstream post-acute referrals from surgical episodes, they may encourage their employed orthopedists to prioritize surgical options. A countervailing force is that employed physicians are typically salaried, which may reduce physician effort, as has been found among some physicians after hospital integration (Post et al., 2023). Whether integration increases or decreases surgical utilization remains an open empirical question.

The second major gap is our weak understanding of how alternative payment paradigms interact with hospital–physician integration. Specifically, the commonly observed effects of such integration – higher spending and higher intensity care – have almost exclusively been studied in fee-for-service contexts. This gap has persisted in part because the majority of care in the United States, particularly hospital-based and specialty care, continues to be delivered under a fee-for-service model (Health Affairs, 2022; Werner et al., 2021). Few settings are amenable to studying alternative payment. Here, we rectify this weakness by taking advantage of a national policy experiment – namely, the Centers for Medicare and Medicaid Services (CMS) Comprehensive Care for Joint Replacement (CJR) model. The CJR model uses bundled payments for hip and knee replacement episodes to incentivize hospitals to coordinate care with other providers in ways that curb spending and improve outcomes from surgery through recovery (Centers for Medicare & Medicaid Services, n.d.-a). CMS randomly assigned hospitals in some metropolitan statistical areas (MSAs) to participate in the CJR program beginning in 2016.

In our study, we exploited this natural experiment by examining the relationship between the CJR program and the treatment pathway of patients with hip or knee osteoarthritis and, critically, assessed whether this relationship varied across competing organizational models (vertically integrated vs. not vertically integrated). Our first objective was to assess whether CJR influenced the osteoarthritis treatment path. The CJR program imposed financial risks on hospitals performing joint replacements. We believed hospitals would be cautious about assuming financial risk for procedures that were previously reimbursed on a fee-for-service basis, so we expected that they might respond to CJR by actually reducing their volume of joint replacement procedures. Second, we sought to assess whether the hospital integration status of the physician directing a patient’s care independently influenced the osteoarthritis treatment path. Because physician discretion influences many decisions along the treatment path, we expected that hospital-integrated orthopedists, ceteris paribus, would direct their patients toward less conservative treatment, that is, away from visits with physicians and toward total joint replacements. Our rationale was that orthopedists managing this care have substantial influence over the patient’s treatment path and that the hospitals employing orthopedists will lean toward more intensive, hospital-based treatments compared with conservative treatments or wait-and-see approaches. We also sought to test whether there was an interaction between CJR and integration. Our reasoning: CJR imposes risks on providers for costly or poorly coordinated care, but this risk is likely smaller among integrated hospital-physician systems. Many have speculated that hospitals and physicians have integrated for exactly this reason – that is, to better coordinate care and respond to alternative payment models (Post et al., 2018). We therefore expect patients of integrated orthopedists to exhibit less CJR avoidance compared with their non-integrated counterparts.

Finally, we sought to test whether CJR affected surgical setting (inpatient vs. outpatient). For certain windows during our study period, outpatient surgeries were exempt from the CJR program. We expected CJR hospitals to shift care to the outpatient setting to avoid the risks of the CJR program.

## New Contribution

This study makes a new and important contribution by examining the relative influence of provider organization and payment models on patient care. We focus on an important type of provider organization, which is the growing trend toward hospital–physician integration, in the context of the bundled payment program that Medicare has adopted for orthopedic services. As U.S. policy makers grapple with different approaches to improving the cost and quality of health care services, this study offers evidence about the influence of two distinct yet potentially synergistic approaches relative to the utilization of an expensive and high-volume orthopedic service.

## Methods

### Data

We used 2015–2023 Medicare administrative claims and Medicare Data on Provider Practice and Specialty (MD-PPAS) data. Claims data included patient diagnoses, medical procedures, enrollment details, demographic characteristics, and the treating physicians, drawn from a nationally representative 5% sample of beneficiaries. MD-PPAS provided information about physicians, including medical specialties and the primary organization in which they practiced. We identified randomly assigned CJR MSAs from CMS documentation and published literature (Supplemental Text 1; Centers for Medicare & Medicaid Services, n.d.-a; Trombley et al., 2022).

### Sample

For each patient in our sample, the care journey began with a new diagnosis of primary or secondary hip or knee osteoarthritis between January 1, 2016, and December 31, 2022. Notably, we did not construct the beginning of an episode using a surgery or other procedure. This is because we were interested in whether or not patients included in the study sample underwent knee replacement. Hip or knee osteoarthritis, which often manifest as discomfort, pain, and limited mobility, is the most common reason for joint replacements among older Americans (Agency for Healthcare Research and Quality, 2025). Osteoarthritis is our “qualifying diagnosis” because it marks the initial step in patients receiving orthopedic care.

All patients received their initial diagnoses between January 1, 2016, and December 31, 2022, so we could verify the absence of a diagnosis in the preceding 12 months (our data begin on January 1, 2015) and track these patients’ care for 12 months post-diagnosis (our data run through December 31, 2023). We used a 12-month post-diagnosis observation window based on our finding that for the study sample, almost 60% of the patients who received a joint replacement within 7 years (i.e., the full length of our data) from the time of their diagnosis of osteoarthritis did so within the first 12 months. Patients who received their joint replacement more than 12 months from the time of diagnosis did so in diminishing numbers for each remaining year of the time frame. As such, we viewed the first 12 months as an important window of observation for examining the influence of hospital–physician integration and payment models on the treatment pathway for patients with lower extremity osteoarthritis. Our results were also robust to using a 24-month timeframe (see Sensitivity Analysis).

We required patients to have at least one visit with an orthopedist, measured using MD-PPAS provider specialty code, within the 12 months following diagnosis. We also required continuous enrollment in Medicare Part A and B for the study period. Patients outside the 50 U.S. states or Washington, D.C. were excluded from the study, as were patients in CJR-ineligible or CJR-voluntary MSAs (Supplemental Table 1). In addition, we excluded osteoarthritis resulting from congenital disorders or traumatic injuries as these conditions may have differential risk for our study outcomes (Supplemental Table 1). This approach allowed us to draw more direct comparisons between our patient groups.

### Variables and Measures

Our exposures were hospital–physician integration (non-integrated vs. integrated) and payment paradigm (non-CJR vs. CJR). We classified orthopedists as hospital-integrated with a method used in the literature that combines a claims-based algorithm developed by Hannah Neprash and colleagues with supplemental data about physicians’ primary places of practice from MD-PPAS (Supplemental Text 2; Neprash et al., 2015; Post et al., 2021; Thai et al., 2023). We attributed patients to the orthopedist with whom they had the most encounters during their 12 months post-diagnosis, a method that follows established attribution approaches (Centers for Medicare & Medicaid Services, 2023). We classified patient care as occurring under the CJR program if their residence was in an MSA assigned by CMS to mandatory participation in the CJR program. CMS initially required 67 MSAs to participate in the CJR program but later revised participation to 34 mandatory MSAs and 33 optional MSAs. For all years of our data, we excluded observations originating in participation-optional MSAs because optional participation violated the random assignment that we sought to exploit for our study. CJR cases came from always-mandatory MSAs, and non-CJR came from the 104 MSAs which CMS assigned as “control” MSAs (MSAs eligible for CJR but randomized to non-CJR).

Our dependent variables included the number of patient visits with primary care physicians (PCPs) and orthopedists, whether patients received a joint replacement, and, among those with a joint replacement, whether they received it in inpatient or outpatient settings. Based on guidelines from major professional societies, there are several clinically recommended treatments for osteoarthritis that physicians may consider, of which joint replacement surgery is just one – there are also pharmacologic (e.g., non-steroidal anti-inflammatory drugs [NSAIDs]) and nonpharmacologic (e.g., weight loss, tai chi, exercise, physical therapy) treatments available (Kolasinski et al., 2020). We measured (via Medicare claims) a visit as an encounter with a primary care physician (specialty codes 1, 08, 11, 38) or an orthopedist (specialty code 20); we counted at most one claim per day to avoid inflating the number of visits in encounters with many claims. We winsorized these count variables at the 99th percentile to address outliers. We identified elective inpatient hip and knee replacements using diagnostic-related group (DRG) codes (primarily 469 and 470 [97% of inpatient surgeries], with robustness checks that excluded the hip fracture versions 521 and 522) and International Classification of Diseases, Tenth Revision, Procedure Coding System (ICD-10-PCS) codes (to determine hip vs. knee). We identified elective outpatient hip and knee replacements using Healthcare Common Procedure Coding System (HCPCS) codes 27130 and 27447.

### Statistical Analysis

In our descriptive analysis, we measured patient demographic and clinical characteristics. Table 1 stratifies these characteristics by patients in CJR versus non-CJR MSAs, testing for differences across groups. Differences between patients of non-integrated and integrated orthopedists are presented in Supplemental Figure 1.

**Table 1.** Characteristics of Patients With Osteoarthritis Diagnoses by CJR Participation Status.

Characteristics	Overall	Control MSAs	CJR MSAs	Standardized mean differences
*N* diagnoses	174,865	112,891	61,974	
Joint type				
Knee diagnoses, *N*	111,894	71,938	39,956	
Knee, %	64	63.7	64.5	0.016
Hip diagnoses, *N*	62,971	40,953	22,018	
Hip, %	36	36.3	35.5	0.016
Year of diagnosis				
2016, %	20	19.9	20.2	0.009
2017, %	16.9	16.8	17.1	0.008
2018, %	14.9	14.8	15.1	0.008
2019, %	14.2	14.5	13.7	0.024
2020, %	10.4	10.6	10.1	0.016
2021, %	11.9	11.9	12	0.005
2022, %	11.6	11.6	11.8	0.007
Female, %	62	61.8	62.4	0.011
White, %	86.6	87.6	84.7	0.083
Black, %	6.9	6.8	6.9	0.002
Hispanic, %	1.4	1	2	0.081
Other/unknown, %	5.2	4.5	6.3	0.079
Age, mean	72.3	72	72.7	0.075
Dual-eligible, %	11.5	10.7	12.9	0.068
Northeast, %	29.7	21.4	44.7	0.512
Midwest, %	18.1	25	5.6	0.56
South, %	37.2	37.6	36.6	0.02
West, %	15	16	13.1	0.083
Charlson score, mean	1.27	1.24	1.31	0.04
History of MI, %	2.9	3	2.9	0.007
Congestive heart failure, %	9.3	9.2	9.6	0.013
Peripheral vascular disease, %	12.4	11.5	14	0.076
Cardiovascular disease, %	7.7	7.3	8.5	0.044
COPD, %	16	15.9	16	0.003
Dementia, %	3.9	3.8	4.3	0.028
Diabetes, %	23.5	22.7	24.8	0.049
Diabetes w/ complications, %	10.9	10.8	11.1	0.009
Renal disease, %	12.5	12.7	12.2	0.016
Ulcers, %	0.9	0.9	1	0.01
Rheumatic disease, %	4.7	4.6	4.9	0.016
Treatment from vertically integrated orthopedist, %	30.7	30.6	30.9	0.005

*Note*. SMD is the absolute difference in proportions (for binary variables) or the absolute difference in means (for continuous variables) divided by the pooled standard deviation. Values below 0.10 indicate adequate balance between groups. Sample: patients with osteoarthritis diagnoses. CJR = Comprehensive Care for Joint Replacement; COPD = chronic obstructive pulmonary disease; MI = myocardial infarction; MSA = metropolitan statistical area; *SD* = standard deviation; SMD = standardized mean difference.

We used several statistical models for our analyses. In all models, we adjusted for key covariates: patient age, sex, race/ethnicity, Medicare-Medicaid dual-eligibility status, original and current reasons for Medicare entitlement (e.g., age, disability, end-stage renal disease), joint type (hip vs. knee), Census region (Midwest, South, West, Northeast), Charlson Comorbidity Index score (for patient clinical severity; Charlson et al., 1987; Quan et al., 2011), and year of diagnosis (for secular trends). In models examining inpatient versus outpatient surgical setting, we also adjusted for hospital inpatient and outpatient surgical volume. We included race/ethnicity and sex because of disparities in accessing orthopedic care (Cavanaugh et al., 2019; MacMahon et al., 2023). We estimated our models with robust standard errors.

We included our independent variables of hospital-integration status and CJR status. We interacted these two variables with each other and with the full set of year variables to allow integration and CJR to vary over time. Because our dependent variables of PCP and orthopedist visits showed overdispersion (variance exceeded the mean) and a right skew, we analyzed these outcomes with a negative binomial model. We analyzed the dependent variable of getting a hip or knee replacement surgery with a linear probability model (with robustness to logit models). For this model, the binary outcome of surgery was 1 if a patient had either an inpatient or outpatient joint replacement and 0 otherwise.

Our third dependent variable was the probability of getting an outpatient joint replacement surgery (vs. inpatient), which we estimated with a linear probability model (with robustness to logit) among the set of patients who received knee or hip replacements. During the study window, Medicare made two changes that we factor into our analysis. First, although joint replacements were historically allowed to be performed only in the inpatient setting, Medicare began reimbursing joint replacements in outpatient settings (knees on January 1, 2018, and hips on January 1, 2020). These outpatient joint replacements were not, at first, subject to the CJR program. Thus, to the extent that CJR hospitals could shift patients into the outpatient setting, they could avoid the CJR program: this is the impetus for measuring whether CJR affected the choice of surgical setting. The second change updated that exclusion: on October 1, 2021, these outpatient surgeries also became subject to CJR. With these policy regimes in mind, we tested whether the surgical setting for knee patients in CJR MSAs was more likely to be outpatient from 2018 Q1 to 2021 Q3, and, for hip patients, from 2020 Q1 to 2021 Q3. We estimated the model effects for knee and hip cohorts separately. We did not include 2016 and 2017 in this model (for knee patients) because there was no variation in the dependent variable over this timeframe (i.e., zero outpatient joint replacements) and likewise dropped 2016–2019 for hip patients. This study was deemed exempt by the corresponding author’s institutional review board.

### Limitations

The study had several limitations that should be noted. First, we lacked detailed clinical data for assessing the severity of patients’ osteoarthritis (ICD-10 codes lack that level of clinical granularity). Patients may self-select toward an integrated (or non-integrated) orthopedist: relatively sicker patients, for example, might opt for hospital-based providers. In observable demographic and clinical characteristics, we found no major imbalances between these two groups, but unobservable self-selection remains possible. Second, we did not examine whether study patients received alternative treatments for osteoarthritis, including tai chi, education, and the use of NSAIDs, because these treatments are either not reimbursed by Medicare or are not reliably documented in administrative claims. Third, our study focused on patients enrolled in fee-for-service Medicare and so study findings may not apply to patients with commercial, Medicaid, or Medicare Advantage insurance. Still, we note that our study included a nationally representative sample of orthopedists, and their clinical practices among traditional Medicare patients likely extend to some degree to patients with other insurance types. Fourth, CMS’s voluntary Bundled Payments for Care Improvement (BPCI) initiative also used prospective bundled payments in orthopedics and operated concurrently with the CJR program. However, we found that during our study period, BPCI participation was low, with fewer than 4% of hospitals in CJR or non-CJR groups participating, suggesting the role of BPCI in our analysis was minimal.

## Results

### Descriptive Results

We identified 174,865 qualifying patient diagnoses: 61,974 (about 35%) were in CJR MSAs and 112,891 (about 65%) in control MSAs. We compared the demographic and clinical characteristics of these two cohorts using standardized mean differences (Table 1).

The randomization mechanism of the CJR program appeared to effectively result in comparable patients between the CJR MSA group and the control MSA group. In control MSAs, 63.7% of the sample had as the qualifying diagnosis osteoarthritis of the knee and the 36.3% the hip; in CJR MSAs, 64.5% of the sample had as the qualifying diagnosis osteoarthritis of the knee and 35.5% the hip. Groups were comparable across race/ethnicity, sex, age, dual eligibility, Charlson scores, and 11 common chronic conditions (e.g., cardiovascular disease). As in prior work, CJR MSAs had more representation in the Northeast whereas control MSAs had more representation in the Midwest (Barnett et al., 2019). CJR MSAs and control MSAs were also comparable with respect to the percentage of patients treated by an integrated orthopedist, each approximately 31%.

### Statistical Model Results

We first estimated our count models (Figure 1). Adjusted for covariates, patients in CJR MSAs received about 0.14 more PCP visits in the 12 months after an osteoarthritis diagnosis compared with those in control MSAs (*p* < .001). This was statistically significant, though small relative to the sample mean of about 6.5 PCP visits. CJR was also associated with 0.23 more orthopedist visits compared with those in control MSAs (*p* < .001). Relative to the sample mean of about 3.5 orthopedist visits, this was about 7% higher. We also assessed the relationship between visit count and vertical integration status. Patients of integrated orthopedists received 0.16 fewer PCP visits compared with those of non-integrated orthopedists (*p* < .001), an effect in the opposite direction of the CJR effect and nearly identical in magnitude. Integration was also associated with 0.59 fewer orthopedist visits (*p* < .001), about 18% lower than the sample mean. Full model results for all statistical models are available in Supplemental Table 2.

**Figure 1.**
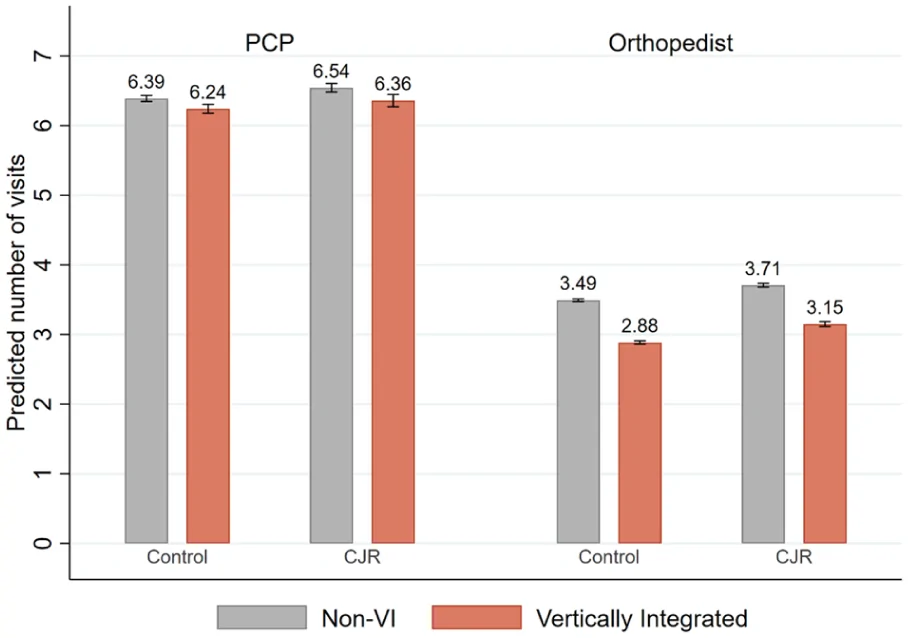
Visit counts by CJR and VI status. *Note*. Predicted counts from negative binomial regressions with CJR × VI × Year interactions, adjusted for joint type and covariates. For PCP visits, the average marginal effect of CJR was 0.139 (*p* < .001) and the average marginal effect of VI was −0.161 (*p* < .001). For orthopedist visits, the average marginal effect of CJR was 0.231 (*p* < .001) and the average marginal effect of VI was −0.590 (*p* < .001). CJR = Comprehensive Care for Joint Replacement; VI = vertically integrated.

We next estimated the effect of the CJR program on the probability of receiving a joint replacement in the 12 months after diagnosis (Figure 2). Throughout the study window, CJR consistently reduced the probability that patients would get a hip or knee replacement. The estimated effects were stable across diagnosis years at approximately −2 pp (average marginal effect −1.9 pp, *p* < .001). The effect size was large relative to the sample mean: 13% of our sample received a joint replacement, implying the CJR program reduced the probability of a joint replacement by about (−1.9/13 = −0.146) 15%. To test whether the CJR effect differed by integration status, we estimated our main model with the full set of interactions between CJR, integration, and study year, and then computed the marginal effects of the CJR program among patients of integrated providers versus patients of non-integrated providers. The effect among non-integrated orthopedists was −1.89 pp and among integrated orthopedists was −1.95 pp; this was not statistically significant (*p* = .39). Furthermore, the association between integration status and joint replacement probability appeared to be consistent across CJR (+1.02 pp) and control (+1.06 pp) MSAs, suggesting that integration exerted a surgery-promoting effect regardless of payment paradigm.

**Figure 2.**
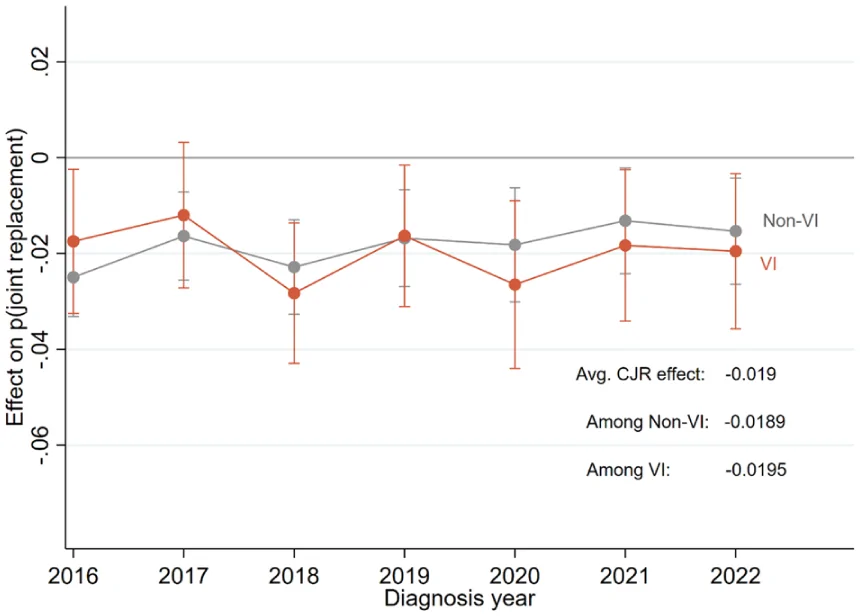
Estimated effects of CJR on joint replacement. *Note*. Linear probability model of the estimated effects of CJR on the probability of a total knee or hip arthroplasty, adjusted for joint type and covariates. Sample-wide probability of surgery: 13.2%. The average marginal effect of the CJR program was −1.9 percentage points (*p* < .001). The difference in the effect of the CJR program among patients of vertically integrated and non-vertically integrated orthopedists was not statistically significant (*p* = .3898). The average marginal effect of vertical integration on surgery probability was 1.0 percentage points (*p* < .001). CJR = Comprehensive Care for Joint Replacement; VI = vertically integrated.

We then estimated our models of surgical setting (binary outcome: outpatient = 1, inpatient = 0), broken out by knee and hip cohorts (Figure 3). Knee patients in CJR MSAs, counter to expectations, did not have higher rates of surgeries in the outpatient setting than those in control MSAs, even during the timeframe in which outpatient surgeries were exempt from the CJR program. In fact, the average marginal effect of the CJR program from 2018 to 2022 on outpatient knee surgery was negative: surgical CJR MSA patients were 4.6 percentage points less likely to get outpatient surgeries than surgical control MSA patients (*p* < .001). Since 48% of surgical knee patients got their surgeries in the outpatient setting (52% inpatient), 4.6 percentage points reflected about a 10% lower chance of outpatient surgery. The hip cohort effects were noisily estimated and not statistically significant (−0.7 pp, *p* = .61; sample mean about 55% outpatient vs. 45% inpatient). The noise may partly reflect that the coronavirus pandemic largely overlapped the timeframe of interest, that is, 2020 Q1–2021 Q3, when hip replacements were outpatient-eligible and exempt from CJR. After the outpatient exemptions to CJR ended on October 1, 2021, we continued to observe negative effects of the CJR program on outpatient setting for knees and noisy effects for hips. Finally, we tested whether the dynamics we observed differed by orthopedist integration status or whether there was an interaction between integration status and CJR status. We found no significant differences between patients of integrated versus non-integrated orthopedists in either cohort (Supplemental Figure 2).

**Figure 3.**
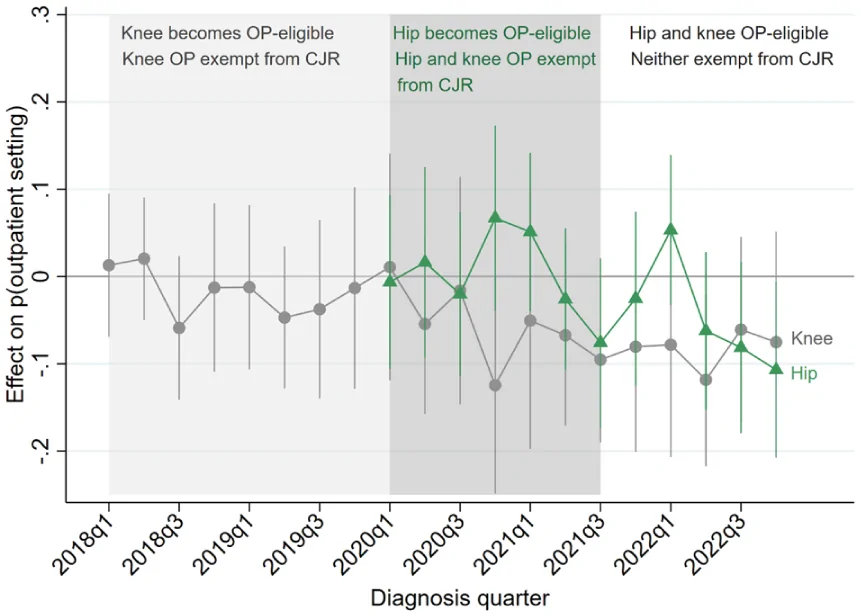
Effects of CJR on outpatient surgical setting. *Note.* Linear probability model of the estimated effects of CJR on the probability of receiving surgery in the outpatient rather than inpatient setting, adjusted for covariates. Shading indicates periods when outpatient surgery was permitted but exempt from the CJR bundle. Knee sample: *N* = 6,627, sample-wide OP rate = 48.1%. Reference quarter: 2017q4 (not plotted). The average marginal effect of CJR was −4.6 percentage points (*p* < .001). Hip sample: *N* = 4,828, sample-wide OP rate = 55.5%. Reference quarter: 2019q4 (not plotted). The average marginal effect of CJR was −0.7 percentage points (*p* = .6072). CJR = Comprehensive Care for Joint Replacement; OP = outpatient.

### Sensitivity Analyses

We tested several alternative approaches to ensure robustness. Estimating our binary models using logit versus our preferred linear probability models showed results that directionally matched and were often very similar in magnitude (e.g., average marginal effect of CJR on surgery of −1.95 pp vs. −1.91 pp, Supplemental Table 2). We estimated a model with a full set of dummy variables for chronic conditions; estimates were nearly identical to the main model that used a summary Charlson score, so we kept the Charlson specification for parsimony. Results were substantially unchanged if we excluded patients under 65; if we excluded patients with both a hip and knee episode; and if we redefined the surgical outcome variable to be surgery within 24 months instead of within 12 months. Our main models used the orthopedist each patient saw the most in the 12 months after diagnosis; we also tested a model in which we used the diagnosing physician’s integration status, which has the advantage of a larger sample size and the disadvantage of including both patients who saw orthopedists (presumably more clinically urgent) and those who did not (presumably less clinically urgent). Results were directionally the same, with most estimates of effect magnitude comparable to our main analysis (Supplemental Figure 3). Our sensitivity analyses confirmed consistency of findings across many analytical choices.

## Discussion

This study examined how hospital–physician integration and bundled payment incentives jointly shape treatment patterns for Medicare patients with hip and knee osteoarthritis by using the random assignment of the CJR program as a natural experiment. CJR was associated with reduced surgical rates and more conservative post-diagnosis management, reflected in higher follow-up visit counts. Vertical integration, by contrast, was associated with higher surgical rates and fewer follow-up visits, consistent with financial incentives that favor surgical volume under fee-for-service. We also found that CJR reduced the likelihood of outpatient surgery, despite outpatient procedures being exempt from the bundle during much of the study period, suggesting unintended effects on surgical setting decisions. Taken together, these findings indicate that alternative payment models and hospital–physician integration can exert opposing and largely independent influences on treatment intensity, and that, at least in the case of lower extremity joint replacements, payment model incentives may eclipse organizational incentives when the two conflict.

Our study adds to published literature on physician integration status and treatment patterns. Some studies have found that in clinical contexts with physician discretion, integration can possibly influence the course of treatment toward more costly or intense care (Harris et al., 2025; Jung et al., 2019; Post et al., 2023). For example, prior work has shown that hospital-integrated PCPs prescribe more inappropriate magnetic resonance imaging (MRI) and that hospital-integrated cardiologists prescribe more cardiac catheterizations and angioplasties (Post et al., 2023; Young et al., 2021). Our results are consistent with this literature: patients of integrated orthopedists had fewer outpatient visits with their orthopedists and PCPs, yet they were more likely to receive joint replacement surgeries.

Our results also inform the literature around the CJR program. We found that patients of orthopedists in the CJR program were significantly less likely to undergo joint replacements and more likely to receive conservative post-diagnosis management, as measured by higher follow-up visit counts. We see this as consistent with our expectation: the CJR program created financial risk for providers, and providers responded by avoiding some of that risk. This is a contribution to the literature, as less surgery was not necessarily CMS’s intent. The incentives CMS created with CJR were focused on containing costs within a 90-day surgery episode. In other words, the CJR program aimed to reduce spending on the intensive margin (lower costs per surgical episode). Our results demonstrate that there was also an extensive margin (fewer surgical episodes in the first place). A full accounting of the cost savings of the CJR program should account for both.

We also explored the intersection of bundled payment and vertical integration. We found no evidence that integration moderated the CJR effect. The surgery-decreasing aspect of CJR was nearly identical whether providers were integrated or not. Similarly, the surgery-increasing aspect of integration was nearly identical whether in control or CJR MSAs. This set of findings implies that integration and payment design largely operated independently of each other – and in our study, they operated at odds. Some prior work has speculated that the consolidation of delivery systems is a reaction to payment reform (Baicker & Levy, 2013; Kocher & Sahni, 2011; Neprash et al., 2017), that is, that hospitals and physicians have merged to better adapt to new payment models, perhaps by allowing hospitals to exert more direction over physician choices in such models. The results of our study do not support that perspective. Integrated providers were mostly indistinguishable from non-integrated providers, suggesting that the strategic advantages of integration under bundled payment may be limited. Instead, we observed that the CJR program counteracted integration. Whereas integration was associated with about 2% fewer PCP visits, CJR was associated with 2% more. While integration was associated with 18% fewer orthopedist visits, CJR was associated with 7% more. And while integration was associated with 8% higher probability of surgery, CJR was associated with about 15% lower probability of surgery.

One unexpected finding was that CJR reduced the probability of surgeries taking place in the outpatient setting. We had expected CJR providers to avoid the financial risk of bundled payments by steering patients into the (temporarily) CJR-exempt outpatient setting; we found the opposite. A partial explanation may be infrastructure: we examined the hospitals in our sample to assess whether there were differences in outpatient capacity by CJR status. In 2018, 61% of control hospitals had outpatient capacity for lower extremity joint replacements (i.e., performed at least one outpatient hip or knee replacement), while only 46% of CJR hospitals did. This gap in outpatient capacity persisted until 2022, when the numbers converged to about 92% (Supplemental Figure 4). Part of the comparatively lower adoption of outpatient surgeries among CJR MSAs could thus be a function of differences in these hospitals’ infrastructure, although since we were unable to assess this in depth, further inquiry is warranted.

## Conclusion

Our study demonstrates that when it comes to managing one of the most common orthopedic conditions among Medicare patients, payment paradigm and vertical integration both shape treatment decisions, but in opposite directions – and, in this case, with payment paradigm apparently the stronger force. Policymakers seeking to reduce costs may find bundled payment to be an effective lever even when applied to organizational arrangements that incentivize higher-intensity care.

## Supplemental Material

sj-docx-1-mcr-10.1177_10775587261468837 – Supplemental material for Hospital–Physician Integration and the Comprehensive Care for Joint Replacement Program Supplemental material, sj-docx-1-mcr-10.1177_10775587261468837 for Hospital–Physician Integration and the Comprehensive Care for Joint Replacement Program by Brady Post, Alexandra Harris, Farbod Alinezhad and Gary Young in Medical Care Research and Review

## Data Availability

Data subject to federal data user agreement.
