# Supplementary material for: Hospital–Physician Integration and the Comprehensive Care for Joint Replacement Program
Source: Med Care Res Rev. 2026 Sep 28;83(6):532–40. doi: 10.1177/10775587261468837 (PMC13740194; doi:10.1177/10775587261468837)
Supplement: Supplementary file 1 [file sj-docx-1-mcr-10.1177_10775587261468837.docx]

**Supplemental Information**

I. **Supplemental Text**
II. **Supplemental Tables**
III. **Supplemental Figures**
IV. **Supplemental References**
I. **Supplemental Text**

**Supplemental Text 1. CJR Policy Supplement**

**What is CJR?**

- The Comprehensive Care for Joint Replacement (CJR) Model is a Medicare Part A and B payment model focused on total hip arthroplasties (THAs) and total knee arthroplasties (TKAs) performed in the inpatient and outpatient setting (Centers for Medicare and Medicaid Services, n.d.).

**CJR episode of care definition**: A CJR episode is defined by the admission of an eligible Medicare fee-for-service beneficiary to a hospital paid under the Inpatient Prospective Payment System (IPPS) that eventually results in a discharge paid under:

- MS-DRG 469 (Major Hip and Knee Joint Replacement or Reattachment of Lower Extremity with Major Complications or Comorbidities (MCC),
- MS-DRG 470 (Major Hip and Knee Joint Replacement or Reattachment of Lower Extremity without MCC),
- MS-DRG 521 (Hip Replacement with Principal Diagnosis of Hip Fracture with MCC), or
- MS-DRG 522 (Hip Replacement with Principal Diagnosis of Hip Fracture without MCC).

To address the removal of TKAs and THAs from the inpatient-only (IPO) list in 2018 and 2020 respectively, CMS changed the definition of an ‘episode of care’, beginning in performance year (PY) 6, to also include outpatient TKA and THA procedures. The episode of care continues for 90 days following discharge from the inpatient hospitalization or the date of the outpatient procedure and includes use of post-acute care (Centers for Medicare and Medicaid Services, n.d.).

**CJR Time Period**: April 2016 – December 2024

- PY 1: April 6, 2016-December 31, 2016
- PY 2: January 1, 2017-December 31, 2017
- PY 3: January 1, 2018-December 31, 2018
- PY 4: January 1, 2019-December 31, 2019
- PY 5.1: January 1, 2020-December 31, 2020
- PY 5.2: January 1, 2021-September 30, 2021
- PY 6: October 1, 2021-December 31, 2022
- PY 7: January 1, 2023-December 31, 2023
- PY 8: January 1, 2024-December 31, 2024 (Centers for Medicare and Medicaid Services, n.d.)

**Which hospitals participate in CJR?**

The CJR model originally required hospitals in 67 randomly selected geographic areas, defined by metropolitan statistical areas (MSAs), to participate. In the third performance year, CMS changed the CJR model so that only hospitals located in the 34 MSAs with the highest historical episode payments were required to continue their participation for the remainder of the model. Hospitals located in a voluntary MSA, or those identified as low-volume or rural, were able to voluntarily elect continued participation in the model by submitting an opt-in letter during the one-time participation election period from January 1, 2018 to January 31, 2018. CMS exempted low-volume hospitals from mandatory participation in CJR; hospitals with 20 or fewer lower extremity joint replacement episodes across the 3 years of historical data preceding the start of CJR were not mandated to participate (Centers for Medicare and Medicaid Services, 42 CFR §510.2). The total number of participating hospitals as of February 1, 2018 was 465; 390 of these 465 hospitals were located in the 34 mandatory MSAs (ten were rural and one was low volume; all 11 opted into continue participation in CJR) while 75 of these 465 providers were located in the voluntary MSAs.

- PYs 1+2: hospital paid under IPPS and located in 67 MSAs
- PYs 3-5: only hospitals in 34 of original 67 MSAs required to participate
- Other hospitals were given one-time opportunity to voluntarily enroll
- PYs 6-8: hospitals in 34 required MSAs that are not designated as low volume or rural

For this study, our CJR exposure group was defined by the 34 mandatory MSAs (see table below), since our outcome period ran from 2016-2021. Hospitals in high-payment MSAs that were eligible but not selected for the CJR model served as our control group (The Lewin Group, 2023).

| **MSA ID** | **MSA Name** |
| --- | --- |
| 10420 | Akron, OH |
| 11700 | Asheville, NC |
| 12420 | Austin-Round Rock, TX |
| 13140 | Beaumont-Port Arthur, TX |
| 17140 | Cincinnati, OH-KY-IN |
| 18580 | Corpus Christi, TX |
| 20020 | Dothan, AL |
| 22500 | Florence, SC |
| 23540 | Gainesville, FL |
| 24780 | Greenville, NC |
| 25420 | Harrisburg-Carlisle, PA |
| 26300 | Hot Springs, AR |
| 28660 | Killeen-Temple, TX |
| 31080 | Los Angeles-Long Beach-Anaheim, CA |
| 31180 | Lubbock, TX |
| 32820 | Memphis, TN-MS-AR |
| 33100 | Miami-Fort Lauderdale-West Palm Beach, FL |
| 33740 | Monroe, LA |
| 33860 | Montgomery, AL |
| 35300 | New Haven-Milford, CT |
| 35380 | New Orleans-Metairie, LA |
| 35620 | New York-Newark-Jersey City, NY-NJ-PA |
| 36420 | Oklahoma City, OK |
| 36740 | Orlando-Kissimmee-Sanford, FL |
| 37860 | Pensacola-Ferry Pass-Brent, FL |
| 38300 | Pittsburgh, PA |
| 38940 | Port St. Lucie, FL |
| 39340 | Provo-Orem, UT |
| 39740 | Reading, PA |
| 42680 | Sebastian-Vero Beach, FL |
| 45300 | Tampa-St. Petersburg-Clearwater, FL |
| 45780 | Toledo, OH |
| 46220 | Tuscaloosa, AL |
| 46340 | Tyler, TX |

**How do the financial incentives of the CJR program work?**

1. CMS provides participant hospitals with a target price for each CJR MS-DRG, prior to the start of each PY. The target price includes a discount over expected episode spending and initially incorporated a blend of historical hospital-specific spending and regional spending for LEJR episodes, with the regional component of the blend increasing over time and eventually being 100 percent regional for PYs 4 through 8.
2. At the end of each PY, actual total spending for the episode is compared to the target price for the participant hospital.
3. Depending on the participant hospital’s quality and episode spending performance, the hospital may receive a bonus payment from Medicare or be required to repay Medicare for a portion of the episode spending.
a. Quality score is based on CMS’ THA/TKA complications and HCAHPS measures; if hospitals submit THA/TKA patient-reported outcomes on a voluntary basis, this can also boost their composite quality score (The Lewin Group, 2023).

**Supplemental Text 2. Determining Hospital-Physician Integration**

We determined physicians' hospital vertical integration status by building on prior work. Specifically, we used tax identifiers, hospital-related keywords, and claims-based approaches. We further refined the keyword approach through manual checks and web searches to reduce Type I and Type II errors.

The data sources we relied on included the Medicare Data on Provider Practice and Specialty (MDPPAS) database from 2013-2021; the American Hospital Association (AHA) annual survey from 2015 and 2020; and the Agency for Healthcare Research and Quality (AHRQ) compendium on health systems from 2016, 2018, and 2022. MDPPAS contains each physician's primary tax identifier number (TIN) and the legal name of the organization from which the physician bills the majority of Medicare services. This is what we mean by the physician’s TIN.

Physicians could qualify as hospital integrated by several different means, which we articulate below. We determined physician integration status separately for each year.

1. **Qualifying based on a TIN legal name connected to a hospital, using AHRQ Compendium**

**Overview**

Hospitals integrate with physicians both directly via employment (e.g., hired directly by Kaiser Permanente) and through acquisitions of independent practices (e.g., Kaiser Permanente acquires Valley Physicians Group, LLC). We searched for both types of physicians in our data. This first method identifies those who were most probably directly employed.

In this method, we created a list of eligible TIN legal names. We identified TIN legal names that we concluded to be hospital TINs during the entire study period. That is, we looked for the TIN names that were quite obviously linked to a hospital. Then, we took this set of TIN names to all the years of our data. For each year that a physician billed the majority of services under a TIN on that list, they qualified as vertically integrated that year.

**Details**

Many hospital systems have different TIN legal names corresponding to different departments or administrative units within the hospital system. We found that one common administrative unit for a hospital was its physician group, under which the large majority (sometimes all) of a hospital’s employed physicians billed for patient care services. For example, the University of Virginia Health System uses the TIN legal name "University of Virginia Physicians Group" for its employed physicians.

To find these hospital TIN names, we conducted a deep dive analysis on the AHRQ Compendium from 2018, roughly the midpoint of our data window. The Compendium comprises between 600 and 700 health systems nationwide and identifies a series of TIN legal names that are associated with each system (a total of about 7,000 TINs). For example, Cleveland Clinic has 14 TINs associated with it, including Cleveland Clinic Florida, Cleveland Clinic Health System - East Region, Cleveland Clinic Nevada, Fairview Hospital, Heart Rhythm Associates, The Cleveland Clinic Foundation, and others. In our list of TIN legal names that we categorized as hospital TINs, we included all those that included the term "Cleveland Clinic" - this covered nearly 90 percent of the physicians that the Compendium reports as part of Cleveland Clinic. Heart Rhythm Associates, by contrast, we did not include in this list because the purpose of this list was to identify TIN names that reliably identified hospitals at all times. The name Heart Rhythm Associates did not obviously tie the organization to Cleveland Clinic.

We expect a very low rate of Type I error from this process, i.e., unless a TIN name was clearly part of the hospital, we avoided classifying it as a hospital TIN. We were particularly careful to avoid greenlighting TINs that could have been the result of practice acquisitions (and would therefore only be hospital-integrated at certain times). For example, University of Pennsylvania Health System had an obvious favored TIN for its physicians (University of Penn Medical Group, with two-thirds of all its physicians as reported in the Compendium). The Compendium also identifies 18 other TINs that are part of the Penn system. Some of these were quite likely the result of acquisitions. For example, "Princeton Family Care" with two physicians was almost certainly the product of Penn acquiring a small primary care practice. Thus, whether looking at data from 2013 or 2021, we were confident that physicians billing the majority of their services under "University of Penn Medical Group" were integrated for purposes of our study. However, we did not make this same assumption for physicians connected to Princeton Family Care. Although the physicians of this practice appear to have been integrated as of 2018 per the AHRQ compendium, we do not have data on when the acquisition was completed. Accordingly, from 2018 onward, it is likely a safe assumption that physicians billing under the Princeton Family Care LLC TIN were hospital-integrated, but the status of such physicians prior to this date is uncertain. As such, having Princeton Family Care as one’s primary TIN name was not ipso facto qualifying as hospital-integrated. We note, though, that this does not preclude the physicians connected with Princeton Family Care from qualifying as hospital-integrated via another approach, e.g., specifically the claims approach described below.

Some systems had ambiguous naming conventions and therefore required manual web searches. For example, when we examined Houston Methodist, there was no TIN that was immediately obvious as their physician group TIN. However, given that one of their TINs ("Tmh Physician Associates, PLLC") comprised nearly 80% of their physicians and Tmh matches the initials for Texas Methodist Houston, we were sufficiently convinced that this name was more likely to be a hospital TIN than a former practice TIN.

Altogether, we identified over 2,000 TIN legal names that we believed were hospital TINs. In any year that most of a physician’s services occurred under one of these, we considered that physician to be hospital-integrated.

2. **Qualifying based on a TIN legal name containing specific keywords**

**Overview**

Method one identified many hospital-based TINs, but hospital names can vary slightly over time. For this reason, we also applied a basic keyword search to each physician’s primary TIN legal name as a backup. If a physician’s primary TIN met the criteria below in any given year, they qualified as integrated in that year.

**Details**

Our review of the AHA data showed that about 80 percent of U.S. hospital names contain the keywords hospital, health system, healthsystem, health science center, health sciences center, or medical center. If a physician’s TIN legal name in MDPPAS contained one of these terms, we flagged the National Provider Identifier (NPI) as possibly hospital-integrated. We assumed those with the word "hospital" were hospital-integrated. The other terms were slightly more ambiguous, especially "medical center" or "health system." “Health system” flagged important hospital systems like Mayo Clinic Health System, but we reviewed the flagged TINs and found that a few were also urgent care clinics or mental health organizations. Thus, we checked the flagged TINs against the AHRQ compendium. If it had a match in the compendium hospital name list, it qualified as integrated; if not, we flagged it for manual web searches. We then removed TINs that were not hospital systems as determined by manual web searches. For example, the legal name "GMG Health Systems Associates, PA" is a medical group, suggested by the "PA" [professional association] suffix and verified via web search. Similarly, though it matches the basic keyword search, "You First Health Systems" is not a hospital but a mental health services organization operating in Maryland.

3. **Qualifying based on a TIN legal name that matched an AHA hospital name**

We compiled a list of hospital names from the AHA data (2015, 2020) and the AHRQ Compendium data (2016, 2018, 2022). We included the names of both individual hospitals and health systems. Physicians working for organizations with TIN names that matched one of these qualified as hospital-integrated.

4. **Qualifying based on claims submission history**

We identified, for each physician in each year, the number of services delivered in either an office or a hospital outpatient department (HOPD) setting, based on Medicare claims. If 75% or more of those were delivered in a hospital outpatient department, the physician qualified as hospital-integrated. We also took steps to reduce errors from this measure. For instance, solo practitioners meeting this criterion qualified as hospital integrated. However, for a TIN with multiple physicians, if one physician was identified as integrated but others connected to the same TIN were not, we flagged this TIN as a potential false positive. We then applied corrections per the following.

Requirements for correcting a physician’s HOPD-billing-based classification of integration:

(1) multiple doctors in the TIN

(2) physician’s claims-based measure of integration was 75% or above, but

(3) three-quarters or more of the other physicians connected to the TIN had claims-based measures under 75%

Under this set of rules, we classified all physicians connected to the TIN as independent, surmising that if three-quarters of a practice appeared to be independent, all associated physicians were likely independent.

We intentionally dropped a few ambiguous cases. Specifically, we dropped the physician-year observations where there was “disagreement” in a TIN about integration status but the TIN did not meet criterion (3) above. Over our 2013-2021 data period, this amounted to about 25,000 (out of more than five million) physician-year observations across all physician specialties. Given the ambiguity, we dropped these observations and our analysis did not include patients attributed to such physicians. We consider the improvement in measurement accuracy worth the negligible sample size reduction.

**Random Sample Checks and Exclusions**

After all of the above, we drew random samples of the data to check for accuracy. We iterated through our process to add exclusions based on what we learned along the way. For example, we had greenlit TIN names containing the term "Spectrum Health" because Spectrum Health is a large hospital system in southwest Michigan. However, from our sample of data we found that a few physicians were connected to TINs with the term "Spectrum Health" in the name (e.g., Full Spectrum Health PLLC, which is not a hospital). We removed the specific keyword “Spectrum Health” from the list we created in method one, but allowed “Spectrum Health Hospitals.” These manual corrections were not common, but contributed to incremental improvements to measurement accuracy nevertheless.

**Advantages and Limitations**

Prior work has used the 75% HOPD claims threshold and the basic keyword search (hospital, health system, medical center, etc.). The approach we have outlined here uses those methods and then reduces measurement error associated with them. In particular, our detailed approach to categorizing hospital TINs reduced important Type II errors (that is, failing to categorize as integrated physicians who in fact were). A basic keyword search would flag systems such as Mount Sinai Health System or Maimonides Medical Center, but it would fail to flag systems whose legal names do not include these terms - e.g., "Cleveland Clinic" and "Kaiser Permanente." We further found that some large systems like the University of California Health System used a wide array of TIN legal names that defied keyword searches: our manual review turned up, for example, "UNIV OF CA" and "UNIVERSITY OF CLAIFORNIA" (typo reproduced). These would have gone unnoticed by keyword searches using correctly-spelled and complete words.

Although our approach is subject to less measurement error than claims-based approaches, it has limitations as well. One limitation may be the result of hospitals or systems changing their names due to rebranding, mergers, or other events. Spectrum Health and Beaumont recently merged to become Corewell Health. This particular event fell outside of our study window but if similar events occurred during our study period, we might have missed physicians who were hired under a legal name of "Corewell Health," as we would not have recognized Corewell Health as a hospital system. Such physicians might still have qualified via the claims-based measure or would have qualified if the AHA data had been promptly updated to include “Corewell Health.” Similarly, some health systems had very nonspecific names such as Memorial Hospital or Community Hospital. In such cases we had to consider the risks of both type I and type II errors. A TIN name such as "Community Physicians" could be Community Hospital's favored TIN for its employed physicians. Adding a rule allowing that phrase would reduce type II errors. However, “Community Physicians” is very ambiguous terminology. Hundreds of independent practices include the phrase "community physicians" in their legal names, which would introduce many type I errors. We therefore avoided adding ambiguous names to the approved hospital TIN list of method one unless we were confident either that there would be few false positives or that we could manually exclude the false positives that such a rule would generate. Last, our random draws for manual verification also helped to increase our confidence in our approach.

II. **Supplemental Tables**

**Supplemental Table 1. Sample Flow**

|  | Resulting sample size | |
| --- | --- | --- |
| Criterion | Patient level | Episode level |
| Total patients with non-congenital or traumatic hip or knee OA diagnosis (2016-2023) | 928878 |  |
| Excluded: first dx in 2015 (failed 12-month lookback) | 753307 |  |
| -- Knee OA only | 427596 |  |
| -- Hip OA only | 200670 |  |
| -- Both knee and hip OA | 125041 |  |
| Excluded: non-US states, incomplete Part A/B coverage, HMO coverage | 616189 | 723924 |
| Restrict to MSAs designated as control or CJR |  | 345206 |
| Drop if diagnosis year=2023 |  | 309778 |
| Restrict to observations with at least one orthopedist encounter |  | 174865 |
| Final analytic sample size |  | 174865 |

Notes: OA – osteoarthritis; CJR – Comprehensive Care for Joint Replacement; MSA – metropolitan statistical area. Sample begins with patient-level construction and converts to episode-level for estimation of statistical models. Sample size for robustness check that uses diagnosing physician vertical integration status of managing orthopedist is n = 278548.

**Supplemental Table 2a. Regression Results – Count Models**

Appendix Table: Regression Results for Figure 1 — Visit Counts by CJR and VI Status

|  | (1) |  |  | (2) |  |  |
| --- | --- | --- | --- | --- | --- | --- |
|  | PCP Visits |  |  | Orthopedist Visits |  |  |
|  | b | ci95 | p | b | ci95 | p |
| CJR MSA | -0.019 | [-0.042,0.004] | [0.100] | 0.040^***^ | [0.020,0.060] | [0.000] |
| Vertically Integrated | -0.009 | [-0.036,0.018] | [0.522] | -0.209^***^ | [-0.232,-0.187] | [0.000] |
| CJR x VI | 0.023 | [-0.022,0.069] | [0.310] | 0.027 | [-0.012,0.066] | [0.181] |
| 2017 (ref.=2016) | -0.043^***^ | [-0.063,-0.023] | [0.000] | -0.025^***^ | [-0.042,-0.008] | [0.005] |
| 2018 | -0.086^***^ | [-0.108,-0.064] | [0.000] | -0.023^**^ | [-0.041,-0.005] | [0.012] |
| 2019 | -0.171^***^ | [-0.194,-0.149] | [0.000] | -0.085^***^ | [-0.104,-0.067] | [0.000] |
| 2020 | -0.138^***^ | [-0.163,-0.112] | [0.000] | -0.067^***^ | [-0.087,-0.046] | [0.000] |
| 2021 | -0.191^***^ | [-0.216,-0.166] | [0.000] | -0.084^***^ | [-0.104,-0.064] | [0.000] |
| 2022 | -0.184^***^ | [-0.210,-0.159] | [0.000] | -0.077^***^ | [-0.096,-0.057] | [0.000] |
| CJR x 2017 | 0.020 | [-0.015,0.054] | [0.261] | 0.043^***^ | [0.014,0.072] | [0.004] |
| CJR x 2018 | 0.061^***^ | [0.024,0.097] | [0.001] | 0.017 | [-0.013,0.048] | [0.270] |
| CJR x 2019 | 0.061^***^ | [0.023,0.100] | [0.002] | 0.034^**^ | [0.003,0.066] | [0.034] |
| CJR x 2020 | 0.038^*^ | [-0.006,0.082] | [0.090] | 0.012 | [-0.024,0.047] | [0.520] |
| CJR x 2021 | 0.091^***^ | [0.049,0.133] | [0.000] | 0.027 | [-0.006,0.060] | [0.112] |
| CJR x 2022 | 0.079^***^ | [0.036,0.121] | [0.000] | 0.004 | [-0.029,0.038] | [0.801] |
| VI x 2017 | -0.021 | [-0.060,0.019] | [0.304] | 0.029^*^ | [-0.004,0.062] | [0.086] |
| VI x 2018 | -0.044^**^ | [-0.084,-0.003] | [0.035] | 0.031^*^ | [-0.003,0.065] | [0.071] |
| VI x 2019 | 0.014 | [-0.027,0.055] | [0.506] | 0.026 | [-0.008,0.060] | [0.127] |
| VI x 2020 | -0.014 | [-0.060,0.032] | [0.559] | 0.032^*^ | [-0.005,0.069] | [0.086] |
| VI x 2021 | -0.023 | [-0.068,0.021] | [0.303] | 0.032^*^ | [-0.004,0.067] | [0.078] |
| VI x 2022 | -0.026 | [-0.071,0.020] | [0.270] | -0.020 | [-0.056,0.016] | [0.275] |
| CJR x VI x 2017 | -0.030 | [-0.097,0.037] | [0.381] | -0.036 | [-0.093,0.021] | [0.211] |
| CJR x VI x 2018 | -0.050 | [-0.118,0.018] | [0.150] | -0.001 | [-0.059,0.057] | [0.973] |
| CJR x VI x 2019 | -0.054 | [-0.125,0.016] | [0.132] | 0.003 | [-0.055,0.062] | [0.906] |
| CJR x VI x 2020 | -0.027 | [-0.107,0.052] | [0.502] | -0.006 | [-0.069,0.058] | [0.859] |
| CJR x VI x 2021 | -0.002 | [-0.076,0.072] | [0.956] | 0.006 | [-0.054,0.066] | [0.855] |
| CJR x VI x 2022 | -0.049 | [-0.125,0.027] | [0.203] | 0.065^**^ | [0.004,0.126] | [0.036] |
| Hip (ref.=Knee) | 0.143^***^ | [0.133,0.152] | [0.000] | 0.055^***^ | [0.047,0.062] | [0.000] |
| Age | 0.018^***^ | [0.018,0.019] | [0.000] | -0.009^***^ | [-0.010,-0.009] | [0.000] |
| Female | 0.088^***^ | [0.079,0.098] | [0.000] | 0.025^***^ | [0.017,0.032] | [0.000] |
| Black | -0.041^***^ | [-0.060,-0.023] | [0.000] | -0.113^***^ | [-0.128,-0.098] | [0.000] |
| Hispanic | -0.141^***^ | [-0.185,-0.098] | [0.000] | -0.015 | [-0.048,0.019] | [0.396] |
| Other Race | -0.020^*^ | [-0.041,0.001] | [0.062] | -0.041^***^ | [-0.058,-0.023] | [0.000] |
| Dual Eligible | 0.224^***^ | [0.208,0.241] | [0.000] | -0.124^***^ | [-0.138,-0.111] | [0.000] |
| Charlson Index | 0.180^***^ | [0.177,0.182] | [0.000] | 0.001 | [-0.002,0.003] | [0.508] |
| OREC: Disability | 0.180^***^ | [0.163,0.196] | [0.000] | 0.006 | [-0.008,0.021] | [0.365] |
| OREC: ESRD | 0.250^***^ | [0.135,0.364] | [0.000] | -0.170^***^ | [-0.269,-0.072] | [0.001] |
| OREC: Disability + ESRD | 0.096 | [-0.022,0.214] | [0.110] | -0.227^***^ | [-0.322,-0.131] | [0.000] |
| CREC: Disability | 0.121^***^ | [0.095,0.147] | [0.000] | -0.106^***^ | [-0.127,-0.086] | [0.000] |
| CREC: ESRD | 0.168^*^ | [-0.008,0.345] | [0.062] | -0.009 | [-0.171,0.153] | [0.913] |
| CREC: Disability + ESRD | 0.256^**^ | [0.046,0.466] | [0.017] | 0.022 | [-0.203,0.246] | [0.849] |
| Midwest | 0.048^***^ | [0.034,0.061] | [0.000] | 0.041^***^ | [0.029,0.052] | [0.000] |
| South | 0.068^***^ | [0.057,0.079] | [0.000] | 0.075^***^ | [0.066,0.084] | [0.000] |
| West | 0.048^***^ | [0.033,0.063] | [0.000] | 0.042^***^ | [0.030,0.053] | [0.000] |
| Constant | 0.100^***^ | [0.049,0.150] | [0.000] | 1.931^***^ | [1.890,1.971] | [0.000] |
| ln(alpha) | -0.492^***^ | [-0.502,-0.482] | [0.000] | -1.380^***^ | [-1.391,-1.370] | [0.000] |
| *N* | 174865 |  |  | 174865 |  |  |

Negative binomial regression coefficients with 95% confidence intervals in parentheses and p-values in brackets. Stars denote significance: * p<0.1, ** p<0.05, *** p<0.01. Outcome variables winsorized at the 99th percentile. Visits measured in the 12 months following initial osteoarthritis diagnosis. Visit counts reflect deduplicated carrier claims (one per provider per date) summed across all providers. Reference categories: Non-CJR (CJR), Non-VI (VI), White (race), Northeast (region), Knee (joint), 2016 (year). PCP model — Avg marginal effect of CJR: 0.139 (p=0.0000); Avg marginal effect of VI: -0.161 (p=0.0000). Ortho model — Avg marginal effect of CJR: 0.231 (p=0.0000); Avg marginal effect of VI: -0.590 (p=0.0000). CJR = Comprehensive Care for Joint Replacement; VI = Vertically Integrated; OREC = original reason for entitlement; CREC = current reason for entitlement; ESRD = end-stage renal disease.

**Supplemental Table 2b. Regression Results – Probability of Joint Replacement Surgery**

Appendix Table: Linear Probability Model Results for Figure 2

|  | (1) |  |  |
| --- | --- | --- | --- |
|  | Probability of Joint Replacement |  |  |
|  | b | ci95 | p |
| CJR MSA | -0.025^***^ | [-0.033,-0.017] | [0.000] |
| Vertically Integrated | 0.013^**^ | [0.002,0.024] | [0.018] |
| CJR x VI | 0.007 | [-0.010,0.025] | [0.390] |
| 2017 | -0.004 | [-0.012,0.003] | [0.269] |
| 2018 | -0.011^**^ | [-0.019,-0.002] | [0.012] |
| 2019 | -0.023^***^ | [-0.031,-0.015] | [0.000] |
| 2020 | -0.024^***^ | [-0.033,-0.015] | [0.000] |
| 2021 | -0.033^***^ | [-0.042,-0.024] | [0.000] |
| 2022 | -0.036^***^ | [-0.045,-0.027] | [0.000] |
| CJR x 2017 | 0.009 | [-0.004,0.021] | [0.169] |
| CJR x 2018 | 0.002 | [-0.011,0.015] | [0.744] |
| CJR x 2019 | 0.008 | [-0.005,0.021] | [0.217] |
| CJR x 2020 | 0.007 | [-0.008,0.021] | [0.357] |
| CJR x 2021 | 0.012^*^ | [-0.002,0.025] | [0.091] |
| CJR x 2022 | 0.010 | [-0.004,0.023] | [0.169] |
| VI x 2017 | -0.013 | [-0.028,0.003] | [0.108] |
| VI x 2018 | -0.007 | [-0.023,0.008] | [0.346] |
| VI x 2019 | -0.005 | [-0.021,0.010] | [0.490] |
| VI x 2020 | 0.007 | [-0.010,0.024] | [0.426] |
| VI x 2021 | 0.003 | [-0.013,0.019] | [0.733] |
| VI x 2022 | 0.006 | [-0.011,0.022] | [0.501] |
| CJR x VI x 2017 | -0.003 | [-0.028,0.021] | [0.803] |
| CJR x VI x 2018 | -0.013 | [-0.037,0.012] | [0.301] |
| CJR x VI x 2019 | -0.007 | [-0.032,0.018] | [0.578] |
| CJR x VI x 2020 | -0.016 | [-0.043,0.011] | [0.254] |
| CJR x VI x 2021 | -0.013 | [-0.038,0.013] | [0.335] |
| CJR x VI x 2022 | -0.012 | [-0.038,0.014] | [0.378] |
| Age | -0.004^***^ | [-0.004,-0.003] | [0.000] |
| Female | -0.017^***^ | [-0.020,-0.013] | [0.000] |
| Black | -0.033^***^ | [-0.038,-0.027] | [0.000] |
| Hispanic | -0.010^*^ | [-0.022,0.001] | [0.084] |
| Other Race | -0.023^***^ | [-0.029,-0.016] | [0.000] |
| Dual Eligible | -0.036^***^ | [-0.041,-0.031] | [0.000] |
| Charlson Index | -0.006^***^ | [-0.007,-0.005] | [0.000] |
| OREC: Disability | -0.028^***^ | [-0.033,-0.022] | [0.000] |
| OREC: ESRD | -0.064^***^ | [-0.092,-0.035] | [0.000] |
| OREC: Disability + ESRD | -0.073^***^ | [-0.102,-0.044] | [0.000] |
| CREC: Disability | -0.057^***^ | [-0.065,-0.049] | [0.000] |
| CREC: ESRD | -0.038 | [-0.083,0.008] | [0.107] |
| CREC: Disability + ESRD | 0.009 | [-0.061,0.080] | [0.791] |
| Midwest | 0.031^***^ | [0.026,0.036] | [0.000] |
| South | 0.018^***^ | [0.014,0.021] | [0.000] |
| West | 0.023^***^ | [0.018,0.028] | [0.000] |
| Hip (ref.=Knee) | 0.103^***^ | [0.100,0.107] | [0.000] |
| Constant | 0.395^***^ | [0.379,0.412] | [0.000] |
| *N* | 174865 |  |  |

Linear probability model coefficients with 95% confidence intervals in parentheses and p-values in brackets. Stars denote significance: * p<0.1, ** p<0.05, *** p<0.01. Outcome: binary indicator for receiving joint replacement within 12 months of diagnosis. N=174865. Sample-wide mean: .132. LPM avg marginal effect of CJR: -.019 (p=0). LPM avg marginal effect of VI: .01 (p=0). Logit avg marginal effect of CJR: -.02 (p=0). Logit avg marginal effect of VI: .011 (p=0). Reference categories: Non-CJR (CJR), Non-VI (VI), Knee (joint), White (race), Northeast (region), 2016 (year). CJR = Comprehensive Care for Joint Replacement; VI = Vertically Integrated; OREC = original reason for entitlement; CREC = current reason for entitlement; ESRD = end-stage renal disease.

**Supplemental Table 2c. Regression Results – Probability of Outpatient Setting**

Appendix Table: Linear Probability Model Results for Figure 3 — Outpatient Surgery Setting

|  | (1) |  |  | (2) |  |  |
| --- | --- | --- | --- | --- | --- | --- |
|  | Knee |  |  | Hip |  |  |
|  | b | ci95 | p | b | ci95 | p |
| CJR MSA | 0.025 | [-0.068,0.118] | [0.599] | 0.103^*^ | [-0.016,0.222] | [0.090] |
| 2018q1 (ref.=2017q4, knee) | 0.022 | [-0.048,0.093] | [0.534] |  |  |  |
| 2018q2 | 0.075^**^ | [0.001,0.148] | [0.047] |  |  |  |
| 2018q3 | 0.058 | [-0.020,0.136] | [0.148] |  |  |  |
| 2018q4 | 0.067 | [-0.013,0.147] | [0.102] |  |  |  |
| 2019q1 | 0.162^***^ | [0.083,0.240] | [0.000] |  |  |  |
| 2019q2 | 0.157^***^ | [0.080,0.234] | [0.000] |  |  |  |
| 2019q3 | 0.185^***^ | [0.100,0.270] | [0.000] |  |  |  |
| 2019q4 (ref.=hip) | 0.123^**^ | [0.029,0.218] | [0.011] | 0.000 | [0.000,0.000] | [.] |
| 2020q1 | 0.270^***^ | [0.182,0.357] | [0.000] | 0.114^**^ | [0.023,0.205] | [0.014] |
| 2020q2 | 0.296^***^ | [0.195,0.398] | [0.000] | 0.155^***^ | [0.058,0.251] | [0.002] |
| 2020q3 | 0.273^***^ | [0.182,0.365] | [0.000] | 0.198^***^ | [0.104,0.291] | [0.000] |
| 2020q4 | 0.351^***^ | [0.256,0.445] | [0.000] | 0.139^***^ | [0.038,0.239] | [0.007] |
| 2021q1 | 0.320^***^ | [0.232,0.408] | [0.000] | 0.162^***^ | [0.070,0.255] | [0.001] |
| 2021q2 | 0.347^***^ | [0.263,0.431] | [0.000] | 0.270^***^ | [0.182,0.359] | [0.000] |
| 2021q3 | 0.412^***^ | [0.327,0.497] | [0.000] | 0.208^***^ | [0.116,0.300] | [0.000] |
| 2021q4 | 0.364^***^ | [0.272,0.457] | [0.000] | 0.273^***^ | [0.171,0.375] | [0.000] |
| 2022q1 | 0.357^***^ | [0.273,0.441] | [0.000] | 0.232^***^ | [0.141,0.324] | [0.000] |
| 2022q2 | 0.426^***^ | [0.346,0.507] | [0.000] | 0.213^***^ | [0.122,0.304] | [0.000] |
| 2022q3 | 0.373^***^ | [0.281,0.465] | [0.000] | 0.261^***^ | [0.169,0.352] | [0.000] |
| 2022q4 | 0.343^***^ | [0.240,0.447] | [0.000] | 0.304^***^ | [0.209,0.398] | [0.000] |
| CJR x 2018q1 | -0.046 | [-0.170,0.077] | [0.462] |  |  |  |
| CJR x 2018q2 | -0.062 | [-0.201,0.077] | [0.381] |  |  |  |
| CJR x 2018q3 | -0.004 | [-0.162,0.154] | [0.960] |  |  |  |
| CJR x 2018q4 | -0.031 | [-0.176,0.115] | [0.679] |  |  |  |
| CJR x 2019q1 | -0.069 | [-0.208,0.070] | [0.329] |  |  |  |
| CJR x 2019q2 | -0.078 | [-0.231,0.076] | [0.321] |  |  |  |
| CJR x 2019q3 | 0.045 | [-0.128,0.217] | [0.613] |  |  |  |
| CJR x 2019q4 | 0.036 | [-0.157,0.229] | [0.715] | 0.000 | [0.000,0.000] | [.] |
| CJR x 2020q1 | -0.102 | [-0.262,0.058] | [0.211] | -0.073 | [-0.252,0.107] | [0.428] |
| CJR x 2020q2 | -0.041 | [-0.227,0.146] | [0.667] | -0.125 | [-0.308,0.058] | [0.181] |
| CJR x 2020q3 | -0.084 | [-0.263,0.095] | [0.358] | -0.079 | [-0.248,0.090] | [0.358] |
| CJR x 2020q4 | -0.097 | [-0.304,0.109] | [0.356] | 0.001 | [-0.182,0.183] | [0.994] |
| CJR x 2021q1 | -0.113 | [-0.274,0.048] | [0.169] | 0.005 | [-0.161,0.171] | [0.950] |
| CJR x 2021q2 | -0.061 | [-0.211,0.089] | [0.423] | -0.100 | [-0.259,0.060] | [0.220] |
| CJR x 2021q3 | -0.114 | [-0.297,0.068] | [0.220] | -0.107 | [-0.276,0.061] | [0.212] |
| CJR x 2021q4 | -0.110 | [-0.299,0.079] | [0.255] | -0.096 | [-0.271,0.078] | [0.279] |
| CJR x 2022q1 | -0.155^**^ | [-0.307,-0.002] | [0.046] | -0.088 | [-0.253,0.076] | [0.291] |
| CJR x 2022q2 | -0.174^**^ | [-0.337,-0.011] | [0.036] | -0.089 | [-0.251,0.073] | [0.282] |
| CJR x 2022q3 | -0.052 | [-0.244,0.140] | [0.593] | -0.171^*^ | [-0.344,0.002] | [0.053] |
| CJR x 2022q4 | -0.069 | [-0.268,0.130] | [0.498] | -0.238^***^ | [-0.416,-0.061] | [0.008] |
| Vertically Integrated | 0.086^*^ | [-0.011,0.183] | [0.084] | 0.050 | [-0.059,0.159] | [0.373] |
| Age | -0.005^***^ | [-0.007,-0.003] | [0.000] | -0.014^***^ | [-0.016,-0.012] | [0.000] |
| Female | -0.035^***^ | [-0.055,-0.014] | [0.001] | -0.079^***^ | [-0.104,-0.055] | [0.000] |
| Black | -0.012 | [-0.061,0.038] | [0.645] | -0.016 | [-0.082,0.050] | [0.637] |
| Hispanic | -0.003 | [-0.091,0.084] | [0.942] | 0.061 | [-0.086,0.208] | [0.417] |
| Other Race | -0.011 | [-0.057,0.035] | [0.640] | -0.044 | [-0.103,0.015] | [0.146] |
| Dual Eligible | -0.080^***^ | [-0.127,-0.033] | [0.001] | -0.143^***^ | [-0.196,-0.090] | [0.000] |
| Charlson Index | -0.032^***^ | [-0.040,-0.025] | [0.000] | -0.046^***^ | [-0.054,-0.037] | [0.000] |
| OREC: Disability | -0.045^**^ | [-0.086,-0.003] | [0.036] | -0.101^***^ | [-0.154,-0.048] | [0.000] |
| OREC: ESRD | 0.497^***^ | [0.345,0.648] | [0.000] | 0.183 | [-0.141,0.507] | [0.268] |
| OREC: Disability + ESRD | -0.241^***^ | [-0.334,-0.149] | [0.000] | -0.070 | [-0.626,0.486] | [0.805] |
| CREC: Disability | 0.007 | [-0.059,0.074] | [0.830] | -0.121^***^ | [-0.205,-0.037] | [0.005] |
| CREC: ESRD | -0.940^***^ | [-1.159,-0.722] | [0.000] | -0.628^***^ | [-0.988,-0.268] | [0.001] |
| CREC: Disability + ESRD | 0.000 | [0.000,0.000] | [.] | -0.405 | [-1.070,0.260] | [0.233] |
| Midwest | 0.074^***^ | [0.043,0.105] | [0.000] | 0.059^***^ | [0.022,0.096] | [0.002] |
| South | 0.087^***^ | [0.061,0.114] | [0.000] | 0.088^***^ | [0.056,0.119] | [0.000] |
| West | 0.099^***^ | [0.065,0.133] | [0.000] | 0.064^***^ | [0.025,0.103] | [0.001] |
| Hospital OP Volume | 0.009^***^ | [0.008,0.011] | [0.000] | 0.010^***^ | [0.009,0.011] | [0.000] |
| Hospital IP Volume | -0.006^***^ | [-0.007,-0.006] | [0.000] | -0.008^***^ | [-0.009,-0.007] | [0.000] |
| Constant | 0.622^***^ | [0.475,0.770] | [0.000] | 1.378^***^ | [1.226,1.530] | [0.000] |
| *N* | 6627 |  |  | 4828 |  |  |

Linear probability model coefficients with 95% confidence intervals in parentheses and p-values in brackets. Stars denote significance: * p<0.1, ** p<0.05, *** p<0.01. Outcome: binary indicator for outpatient surgery setting. Knee: N=6,627, sample-wide mean OP rate: 0.4814. Hip: N=4,828, sample-wide mean OP rate: 0.5551. Knee — LPM Average Marginal Effect of CJR: -0.0461 (p=0.0001). Logit Average Marginal Effect: -0.0369 (p=0.0009). Hip — LPM Average Marginal Effect of CJR: -0.0073 (p=0.6072). Logit Average Marginal Effect: -0.0063 (p=0.6424). Knee reference quarter: 2017q4. Hip reference quarter: 2019q4. Reference categories: Non-CJR (CJR), Non-VI (VI), White (race), Northeast (region). CJR = Comprehensive Care for Joint Replacement; VI = Vertically Integrated; OREC = original reason for entitlement; CREC = current reason for entitlement; ESRD = end-stage renal disease.

I. **Supplemental Figures**

**Supplemental Figure 1. Characteristics of Patients Treated by Non-Integrated and Vertically Integrated Orthopedists**

**Supplemental Figure 2. Outpatient Setting Results by VI Status**

**Supplemental Figure 3a. Sensitivity Analysis – Diagnosing Physician VI Status, Count Models**

Note: Estimates here are substantially similar to our main model, where we find positive effects in the CJR program and negative effects for vertical integration, all statistically significant. Estimates here are slightly larger in magnitude.

**Supplemental Figure 3b. Sensitivity Analysis – Diagnosing Physician VI Status, Joint Replacement Probability**

Notes: These estimates are directionally similar to our main model, and the estimate of the effect of CJR among non-VI orthopedists’ patients is very close in magnitude (-1.8pp here, -1.9pp in our main model). Here, the effects of CJR among VI patients is closer to 0. We have more trust in the estimates of our main model because all such patients consulted with an orthopedist after their osteoarthritis diagnosis (and 13% went on to get surgery), while here any patient with a mere diagnosis – from any physician type, including PCPs – is used for estimation (only 8% went on to get surgery). Our main sample is therefore likely to be comprised of patients who are more clinically homogeneous, i.e., all people with osteoarthritis of sufficient complexity to warrant meeting with an orthopedist. We present these sensitivity analyses for thoroughness and transparency, and we note that diagnosing physicians themselves present a clinically interesting concept, as many decisions are likely directly downstream of an initial diagnosis. Their role warrants further inquiry beyond our scope here.

**Supplemental Figure 3c. Sensitivity Analysis – Diagnosing Physician VI Status, Outpatient Setting Probability**

Note: Estimates here are very similar to our main model, where the effect among knee patients is negative with a magnitude of about 4.5 percentage points and the effect among hip patients is not statistically distinguishable from zero.

**Supplemental Figure 4. Outpatient Capacity by CJR Status**

Note: A provider-year observation takes the value of 1 if it had any reported hip or knee replacement surgery and 0 otherwise, drawn from the Medicare outpatient claims file. The observations presented here are limited to the subset of providers that treated patients in the main analytical sample.

**IV. References**

Centers for Medicare and Medicaid Services. n.d. Comprehensive Care for Joint Replacement Model. <https://www.cms.gov/priorities/innovation/innovation-models/cjr>

Centers for Medicare & Medicaid Services. 42 CFR § 510.2: Comprehensive Care for Joint Replacement Model — Definitions. Published November 24, 2015; last amended May 3, 2021. Available at: <https://www.ecfr.gov/current/title-42/chapter-IV/subchapter-H/part-510/subpart-A/section-510.2>

The Lewin Group. (2023). CMS Comprehensive Care for Joint Replacement Model: Performance year 5 evaluation report. https://www.cms.gov/priorities/innovation/data-and-reports/2023/cjr-py5-annual-report
